# Endoscopic dacryocystorhinostomy

**DOI:** 10.5935/1808-8694.20120043

**Published:** 2015-10-20

**Authors:** Renato Roithmann, Tiana Burman, Peter-John Wormald

**Affiliations:** ^1^ Professor of Otorhinolaryngology - Brazilian Lutheran University. Associate Scientific Staf Department of Otolaryngology H & N Surgery - Mount Sinai Hospital Toronto Canada. Head of the ENT Department - ULBRA/Mãe de Deus University Hospital; ^2^ Ophthalmologist, expert in ocular plastc surgery; PhD in Ophthalmology - University of São Paulo; Preceptor at the Ophthalmology Residency Program - Santa Casa de Misericordia de Porto Alegre; ^3^ Professor and Chairman Department of Otolaryngology Head and Neck Surgery, University of Adelaide, Adelaide, Australia. Chairman - Department of ORL-HNS - University of Adelaide. Universidade Luterana do Brasil, Canoas, Rio Grande do Sul e University of Adelaide, Adelaide, Australia

**Keywords:** dacryocystorhinostomy, lacrimal apparatus diseases, natural orifice endoscopic surgery

## Abstract

The endonasal surgical approach of the lacrymal sac assisted by video-endoscopy is carried out today with high success rates. Despite the satisfactory results reached with the traditional external approach, it has the disadvantage of requiring a skin incision and a consequent local scar. With the development and enhancement of the endonasal techniques, the endoscopic approach is increasingly preferred by surgeons.

**Objective:**

This paper reviews the lacrymal system anatomy, the preoperative assessment and the technical details of the endoscopic assisted approach which may provide better surgical outcomes for patients. We will also briefly discuss complications and causes for surgical failure.

**Methodology:**

This is a review of the experience of the authors in the past 10 years of employing the endoscopic technique for the lacrymal sac surgery.

**Conclusion:**

Outcomes regarding the endoscopic dacryocystorhinostomy are, at leas, equal to those from the traditional external approach. Notwithstanding, the joint work between the otorhinolaryngologist and the ophthalmologist is of great benefit to patients with epiphora.

## INTRODUCTION

Dacryocystorhinostomy (DCR) is the standard treatment for nasolacrimal duct obstruction^1^. Based on opening the lachrymal sac, which is connected to the nose, by removing the bone and the mucosa between these two structures at the level of the middle meatus. The traditional technique-of-choice by ophthalmologists is the external approach, in which an incision is made on the skin in order to access the bone, followed by an external osteotomy, opening the nasal mucosa and creating the lacrimal sac flaps from outside to the inside.

The endoscopy-assisted endonasal approach follows the inverse pathway. A nasal mucosa flap is first created, followed by endonasal bone osteotomy to expose the lacrimal sac and its marsupialization to inside the nasal cavity. The endoscopic exposure and view of the entire lacrimal sac is simply fantastic. Success rates of this procedure by both approaches, the external and the endoscopic one, are higher than 90% in seasoned hands.

The joint work between the ophthalmologist and the otorhinolaryngologist is very beneficial to the patient. The ophthalmologist is responsible for the differential diagnosis of epiphora (see irrigation test), as well as for indicating the surgery, the concomitant treatment of the obstruction points and lacrimal canaliculi when present, and intraoperative probing. The otorhinolaryngologist is then responsible for the pre-operative diagnosis of associated nasal disorders (i.e. Obstructive deviation of the nasal septum, nasal conchae hypertrophy, synechia, polyps, chronic rhinosinusitis, and others) and the concurrent intraoperative treatment of these disorders when present, besides exposing, opening and making the lacrimal sac flaps assisted by nasal endoscopy. Postoperative care must be carried out by both, ophthalmologist and otorhinolaryngologist, until complete healing is achieved and the lacrimal pathway is patent and working.

### Objectives

To revise the most relevant aspects associated with endoscopic dacryocystorhinostomy, from the preoperative assessment all the way to the postoperative care for a proper rehabilitation of the lacrimal pathway. To briefly review the lacrimal sac anatomy and preoperative assessment of patients with epiphora.

## METHODOLOGY

Review based on evidence collected by the authors in the past 10 years of using video endoscopy to perform endonasal dacryocystorhinostomy.

### Interesting background

In 1893, Caldwell, an otorhinolaryngologist, described the endonasal approach to the lacrimal sac^2^. Problems especially associated with instrumentation prevented a proper visualization of the structures, and the technique was abandoned.

### External dacryocystorhinostomy

The basis of modern dacryocystorhinostomy has been assigned to Toti, who described, in 1904, the lifting of the lacrimal sac, with the perforation of the adjacent bone, maintaining intact the nasal mucosa^3^, ^4^. Later on, Kuhnt suggested the making of a nasal flap, suturing the adjacent bone periosteum. Ohm developed an instrument used to suture the nasal mucosa flaps and the lacrimal sac, a technique which was later popularized by Dupuy-Dutemps and Bourget, which started to yield high success rates^3^, ^4^.

Concerning the intubation of the lacrimal path, Graue & Glenie were the first to suggest this possibility, utilizing a silver wire^4^, ^5^. In 1950, Henderson, suggested the use of a polyethylene tube^4^, ^6^ and Huggert was the first to describe bicanalicular intubation with polyethylene tubes, in 1959^4^, ^7^. In 1966, Bjork described the bicanalicular intubation in endoscopic dacryocystorhinostomy, which was limited to cases of canalicular disorders and reoperations^4^, ^8^. In 1967, Gibbs introduced the silicone tube, which represented a major progress in lacrimal passage surgery, because it is the least traumatic to the adjacent tissue, including the cornea^4^, ^9^.

The outcome of the external dacryocystorhinostomy in experienced hands, both anatomical and in ameliorating the symptoms of epiphora is very satisfactory, oscillating around 90%-95%^10^, ^11^.

### Endoscopic dacryocystorhinostomy

The first series of endonasal endoscopic procedures were published in the late 80‘s and early 90‘s^12^, ^13^, ^14^. Nonetheless, outcomes comparable to the external technique were only reached when we started removing the bone and achieving full exposure of the lacrimal sac^15^, ^16^. The initially described endoscopic approach led to the opening of the lacrimal sac on the lateral wall, just anterior to the middle concha. As soon as it was shown that the uppermost portion of the lacrimal sac extended about 8 mm above the middle turbinate insertion on the lateral wall, and not only anterior to it^15^, it became possible to make the mucosal flap and consequent complete marsupialization of the lacrimal sac. Thus, the results of such procedure became comparable to those from the external approach^17^.

Although the endonasal endoscopic approach is the one most used today, famous ENTs such as Heermann, in 1958, used the surgical microscope with good outcomes also with the endonasal approach^18^.

### Anatomo-physiology of the lacrimal system

**Figure 1:**
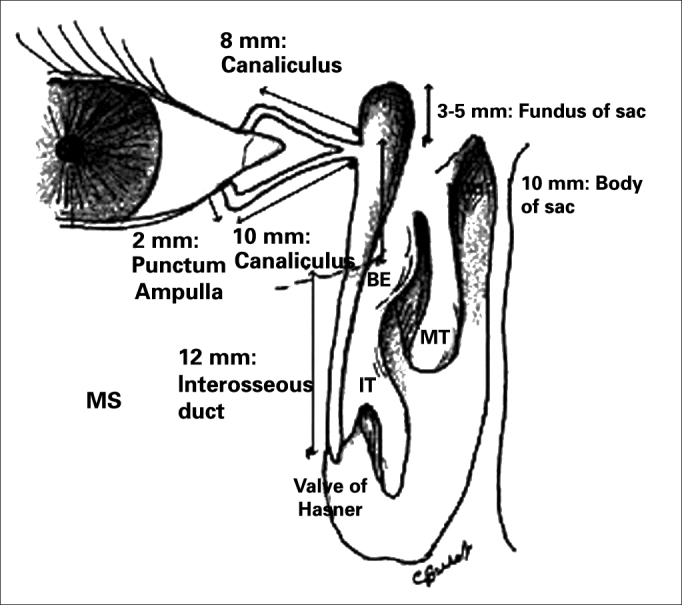
IT: Inferior Turbinate, MT: Middle Turbinate, BE: Ethmoidal Bulla. Notice that the bottom of the lacrimal sac extends superiorly to the middle concha insertion on the lateral wall (about 8 mm above).

Source: Burkat CN, Lemke BN, Anatomy of the orbit and its related structures. Otolaryngol Clin N Am 38:825-56;2005^19^.

Figure 1 illustrates the anatomy of the lacrimal system^19^.

The lacrimal system has the secreting and excreting systems. Tears are produced by the secreting system and drained by the excreting system. The Krause and Wolfring accessory lacrimal glands, located in the conjunctival fornices, are responsible for the basic secretion, while the main lacrimal gland, located in the lacrimal gland fossa of the frontal bone, is responsible for the reflexive production of tears, caused by an irritative or emotional mechanism^20^.

**Figure 2:**
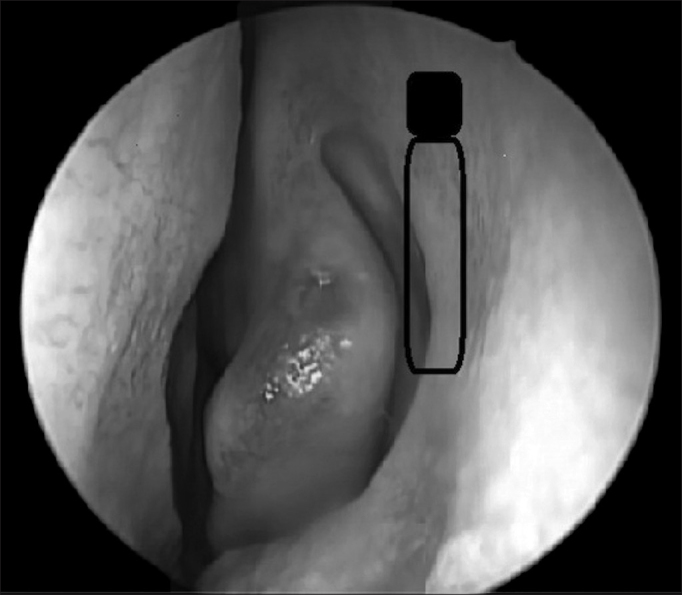
Endoscopic view of the left-side nasal cavity. The black rectangle shows the preciously accepted incorrect extension of the lacrimal sac. The shaded area shows the correct position with its superior extension in average 8 mm above the middle concha insertion.

The excretory lacrimal system drains tears through the upper and lower lacrimal points, superior and inferior canaliculi, common canaliculus, lacrimal sac and lacrimal duct, all the way to the inferior meatus in the nose (Figure 2). The blink reflex also plays an important role in the lacrimal flow is the so-called lacrimal pump mechanism^20^.

The inferior lacrimal point is more easily seen when one inverts the eyelid margin. It is located more laterally vis-à -vis the superior one. The canaliculi continue from the lacrimal points and are made of an initial vertical short portion, and a longer and more horizontal portion follows parallel to the eyelid margin (Figure 1)^20^. At about 1-2 mm short of penetrating the lacrimal sac, the two canaliculi fuse, forming the common canal.

The lacrimal sac has an oval shape, measuring in average 14mm in height and 10mm in width. The lacrimal fossa is formed posteriorly by the lacrimal bone (thin, fragile and easily removed). Both portions fuse in a vertical suture, which may be surgically identified through the endoscopic approach. From its inferior portion, stems the bony nasolacrimal canal, which ends in the inferior meatus, at about 10 mm posterior to the lower turbinate. The lacrimal canal opens up and releases the tear inside the lacrimal sac, in the upper third with inferior two-thirds portion.

One very relevant anatomical detail for the endonasal surgeon is to recognize the correct position of the lacrimal sac on the lateral wall, knowing that the surgical outcome depends totally on its opening. The basic landmark may be the middle concha, and the lacrimal sac is anterior on the lateral wall and it extends, in average, 8mm above the middle concha junction (middle concha insertion point on the lateral wall) (Figure 2).

### Preoperative assessment of patients with epiphora

Tearing is what we call an excess of tears caused by irritation (conjunctival disease, blepharitis, eyelid malpositioning, and others). Epiphora is the tearing that happens secondary to obstruction of its draining system: stenosis or obstruction of the lacrimal point(s) and/or canaliculi or nasolacrimal duct block.

Patients with dysfunctions in the excretion system of the lacrimal path may have epiphora, secretion and a mass in the medial corner of the lacrimal sac. Epiphora alone, without any other symptom suggests the obstruction or malpositioning of the lacrimal point or canaliculus. Secretion suggests obstruction of the nasolacrimal duct, caused by stasis in the lacrimal sac. A mass in the medial sac deserves a careful assessment, since it may be seen in a number of disorders, from abscesses all the way to a tumor in the lacrimal sac. It may also happen in cases of mucoceles, hemangiomas and meningoceles^20^. A positive expression of the lacrimal sac, consisting of secretion reflux via the canaliculi when pressure is applied to the lacrimal sac, is a pathognomonic sign of nasolacrimal duct obstruction. In the epiphora caused by nasolacrimal duct obstruction, the tears frequently run down the patient's cheeks.

The irrigation test via the inferior canaliculus is very simple and may be done in the office. It is based on injecting saline solution with the help of a cannula and a syringe via the inferior canaliculus. Its reading depends whether or not the solution goes through to the oropharynx, associated with reflux or not through the superior or inferior canaliculus. Table 1 shows the many possibilities of outcomes in the test of inferior canaliculus irrigation and its association with the diagnosis at the site of lacrimal path obstruction.

**Table 1:** Preoperative diagnosis of the obstruction site by the irrigation test.

Flushing through the inferior canaliculus	Refux	Nasolacrimal duct
Positive to the oropharynx	No	Normal
Positive to the oropharynx	Through the superior canaliculus	Partial obstruction of the nasolacrimal duct
Negative to the oropharynx	Superior canaliculus with secretion	Complete distal obstruction of the nasolacrimal duct
Negative to the oropharynx	Superior canaliculus without secretion	Obstruction of the common canaliculus
Negative to the oropharynx	Through the inferior canaliculus	Obstruction of the inferior canaliculus

It is very important to have a preoperative diagnosis of stenosis or narrowing, for instance, of the canalicular system, because in such cases, besides the possibility of correcting it in the same dacryocystorhinostomy procedure, the patient must be told of the possibility of a worse surgical outcome. Therefore the ophthalmologist has a very important role to play in the clinical assessment, differential diagnosis and, especially, concerning surgical indication.

Dacryocystography is a diagnostic test that is very much utilized to pinpoint the site of obstruction, in which a contrast medium is injected through the canaliculus and a radiography is taken. Usually, when we have a complete obstruction of the nasolacrimal duct, we find an upstream dilatation of the lacrimal sac^21^. Contrast medium failure in penetrating the lacrimal sac may indicate obstruction of the common canaliculus. Should the contrast pass easily through the nasolacrimal duct, this may indicate a functional problem instead of an anatomical one. The test is contraindicated in the presence of acute dacryocystitis. The assisting clinician must remember that, because of canalicular intubation and the active contrast injection, the site of stenosis may be bypassed. The test must, then, be correlated with clinical findings.

Other tests which may be carried out are the lacrimal system scintigraphy, which may help in the diagnosis of these cases of functional, and not anatomical disorders^21^. Eventually, a CT scan may be ordered in order to assess neighboring structures (anterior orbit, paranasal sinuses, etc.).

The endoscopic examination of the nasal cavity is also important in the preoperative evaluation. It is very common to have a high deviation in the nasal septum which prevents work in the middle meatus. Other diagnosis also happen, such as synechia, polyps, chronic rhinosinusitis and, in children, adenoid hypertrophy. One of the advantages of the endoscopic nasal surgery is the very possibility of simultaneously correcting associated nasal problems. Thus, ENT assessment is also an important part of the pre-op of patients with epiphora.

The preoperative etiological diagnosis of epiphora is very important, since dacryocystorhinostomy is not the indicated treatment for all the cases. Lacrimal point stenosis, the eyelid ectropion or that of the lacrimal point and eyelid laxity are causes of epiphora which are not treated by dacryocystorhinostomy. We must also bear in mind that for cases of canalicular obstruction, especially when proximal, conjunctival dacryocystorhinostomy is indicated with the placement of the Jones tube, which is a non-removable acrylic tube.

### Endoscopic Dacryocystorhinostomy – technical details

#### Preoperative care

Preoperative care is similar to that used in endoscopy-assisted surgery in other nasosinusal disorders^22^. The patients must be instructed to suppress the use of aspirin or other non-hormonal anti-inflammatory agents for at least 7-10 days before the surgery. Other drugs which change the blood coagulation (e.g. Ginkgo biloba, anti-platelet agents and other anticoagulants) must also be suppressed. In the presence of acute nasosinusal inflammatory process, one should use antibiotics and oral steroids. The risks and benefits of the surgery must be discussed, as well as the need for periodic postoperative reviews. Patients must sign the informed consent form. Always bear in mind the use of the silicone tube (Silastic, and others) in the lacrimal pathway, and it's removal, in average, one month after the procedure.

#### Intraoperative technical details

The procedure can be carried out with the conventional instruments used in sinonasal endoscopic surgery. Practically, every surgery is assisted by a 0° wide angle scope. Nevertheless, the use of advanced instruments such as the Microdebrider coupled to flushers and specific tips (delicate straight or angled burr tips) really makes it easier to do the endoscopic procedure.

Local anesthesia monitored by an anesthesiologist or general anesthesia can be used according to the experience of the surgical team. When carried out under general anesthesia, total intra-venous anesthesia is used with propofol and remifentanyl, which usually causes less intraoperative bleeding. Ideally, the heart rate must be around 60-65 bpm and the mean arterial pressure between 60 and 75 mmHg.

The patient's eye must be uncovered on the operated side, avoiding using ointments. Use frequent flushings with saline solution.

Topical vasoconstriction of the nasal mucosa can be achieved by applying cotton rolls soaked in a solution of adrenaline diluted in saline solution at 1:2000. The cotton rolls are inserted in both faces of the middle conchae. It is also a routine to inject lidocaine with a vasoconstrictor agent in the insertion of the middle conchae (anterosuperior on the lateral wall).

Regional septoplasty must be carried out when the nasal septum prevents or hampers the work in the operative field.

**Figure 3:**
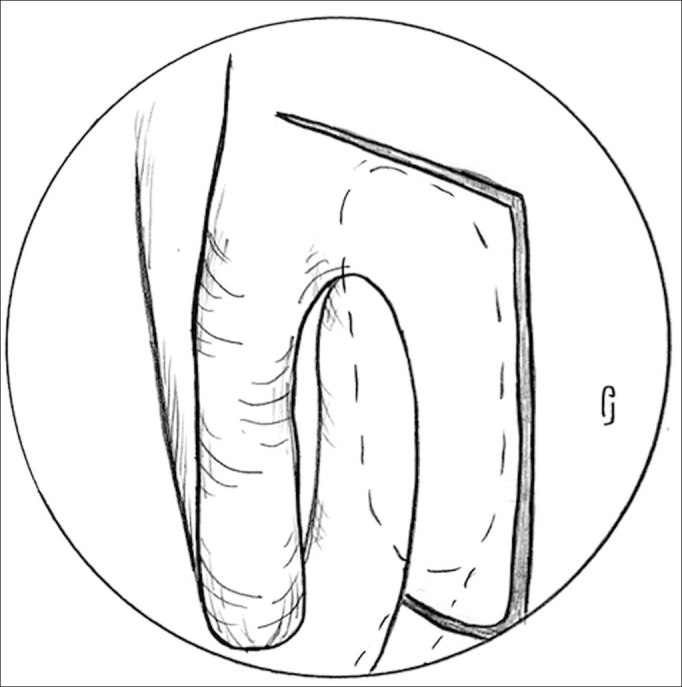
Incisions to make the nasal mucosa flap - Notice the 8 to 10 mm incision above the middle concha insertion point, starting at about 3mm posterior to the insertion point and progressing anteriorly until about 10mm over the frontal process of the maxilla. The vertical incision extending all the way up to the 2/3 of the middle concha height and the last horizontal incision, from the unciform apophysis until it meets the vertical incision.

The next step is to make the mucosal flap on the lateral wall in order to expose the lacrimal fossa bone. For this step we use a conventional scalpel with a 15 blade and the Neves Pinto aspirator/lifter. The first incision is horizontal and made at 8 to 10 mm above the middle concha insertion point, starting about 3mm posterior to the insertion and moving on anteriorly until about 10mm over the frontal process of the maxilla (bony portion of the lateral wall, anterior to the middle concha). Following that, we make a vertical incision extending until the 2/3 of the middle concha height, stopping above the insertion of the inferior concha on the lateral wall. And, finally, we make a new horizontal incision, from the unciform apophysis until it meets the vertical incision (Figure 3).

**Figure 4:**
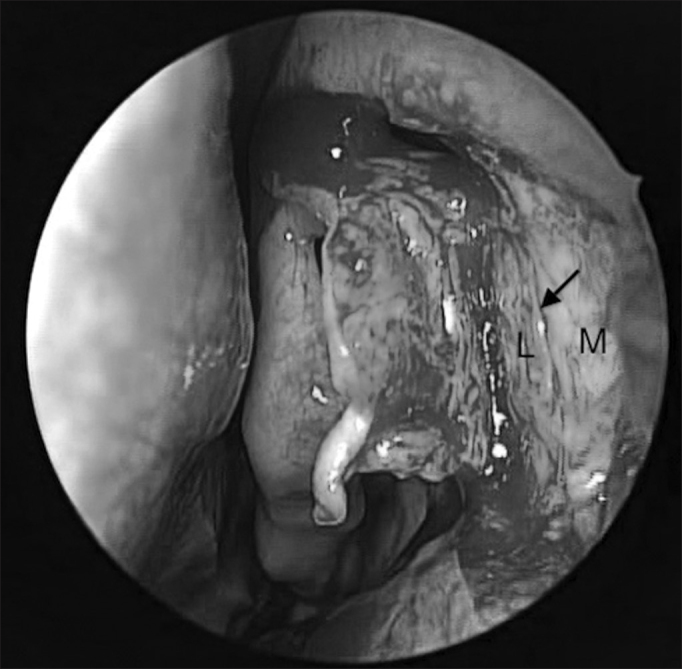
The arrow points to the vertical line separating the frail lacrimal bone (L) from the consistent frontal process of the maxillary bone (M). Notice the opening of the Agger nasi above the middle turbinate insertion point.

Following that, we raise the mucosal flat, keeping it always in contact with the bone (Figure 4).

One key anatomical landmark in exposing the lacrimal fossa is the junction of the hard portion of the frontal process of the maxilla (lateral and anterior) with the soft portion of the lacrimal bone (medial and posterior) (Figure 4). The thin lacrimal bone has a mean width of 2 mm to 5 mm and it is located anterior to the insertion of the unciform apophysis on the lateral wall.

A Rosen lifter, utilized in ear surgery, or even a delicate sickle knife, can be used to separate the most frail portion from the stronger one and, then, start exposing the most medial and posterior wall to the lacrimal sac.

**Figure 5:**
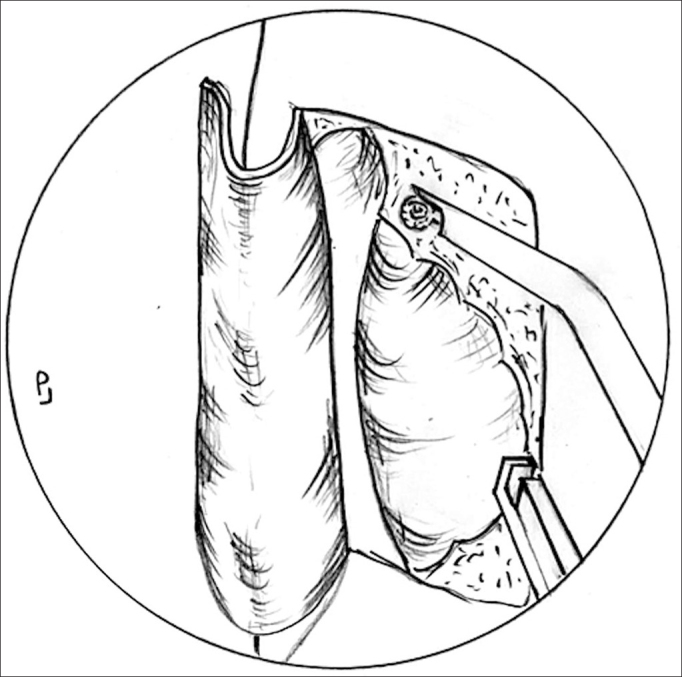
Lacrimal bone removal - Lacrimal bone removal is usually carried out with the Kerrinson forceps and/or the Microdebrider with diamond or cutting burrs (superior and anterior stronger portions of the bone.

At this point, a Kerrinson or Hajek Koeffler forceps is utilized to start removing the stronger portion of bone in the lacrimal fossa (frontal process of the maxilla) (Figure 5). One has to be careful not to damage the anterior wall of the lacrimal sac at this point, since the forceps will apply pressure at this level. The bone is removed as much as possible throughout the entire lacrimal fossa. The anterosuperior most portion is usually very hard, and for that one must use a scope or cutting burrs or, preferable, diamond burrs. The burring systems commonly used in ear surgery may be also used here. Should this be the case, one must be very careful to protect the burr handle and tip so as to avoid burns and consequent scar shrinkage in the nasal vestibule area. If possible, one should use the diamond burr Microdebrider (2.9 mm with a 15° curvature from Medtronic Xomed), it is very useful since, besides being very efficient, it has a flushing system coupled to it, which facilitates and reduces the time needed to perform the procedure (Figure 5).

**Figure 6:**
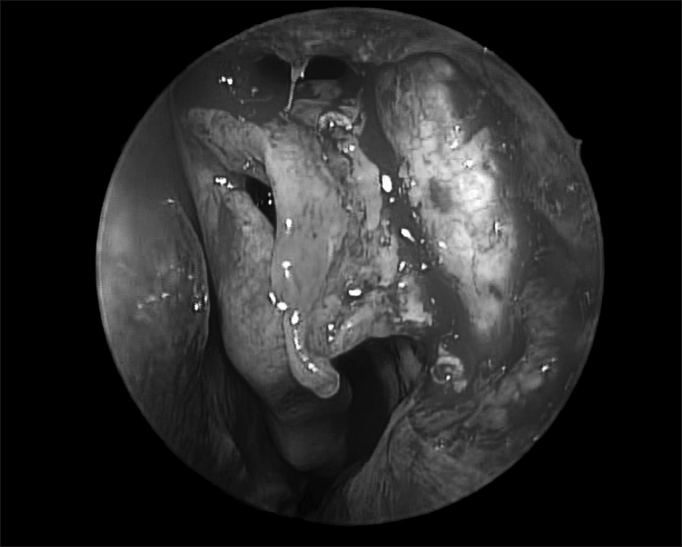
Exposed lacrimal sac (notice the whitish color and the Agger nasi opening above the middle concha insertion point).

The bone opening should be as large as the mucosal opening, which will enable complete visualization of the lacrimal sac (a white color) (Figure 6). Although still controversial, the small osteotomy size has been considered by some studies as a common cause of DCR failure^23^, ^24^.

In many a case, the anterior most ethmoidal cell, the Agger nasi, will be exposed during removal of the most superior and medial portion of the lacrimal fossa bone (Figure 6).

**Figure 7:**
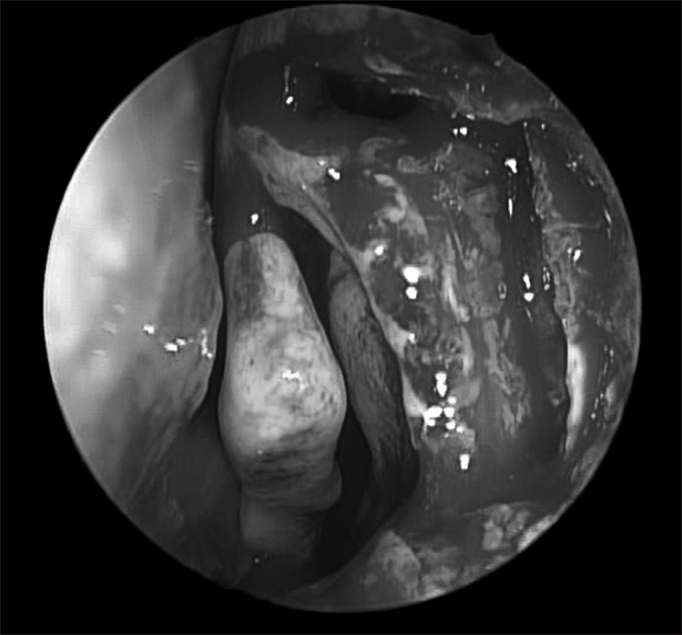
Notice that the posterior flap of the lacrimal sac is in direct contact with the mucosal flap initially made. The lacrimal sac anterior flap is anteriorly rotated over the remaining maxillary bone.

The next step is the dilatation of the lacrimal point and lacrimal pathway catheterization. The Bowman canal probe can be utilized and it will help stretch the lacrimal sac medially, making it easier to cut it with a delicate sickle knife. It is important to visualize the tip of the probe moving freely inside the lacrimal sac. After opening the lacrimal sac longitudinally in its entire extension, two flaps are made. If possible, the anterior should be larger, since it will be turned over the remaining bone of the frontal process of the maxilla. This is made possible by making the longitudinal incision of the lacrimal sac a little more posterior in relation to the midline. The posterior flap will remain in direct contact with the initially-made mucosal flap (Figure 7). For this to happen, the mucosal flap is partially resected with a cutting forceps, until it gets to the level of the posterior flap of the lacrimal sac. The upper portion of the mucosal flap may be repositioned over the middle concha insertion point in order to cover any portion of the remaining bone at this level - should that be the case. The mucosa which covers the most lateral wall of the Agger nasi will also be juxtaposed to the medial wall mucosa of the lacrimal sac flap. By doing it this way we avoid leaving bone exposed in the entire operating field, thus benefitting postoperative healing.

To insert a silicone tube (e.g.: Silastic, Crawford probe, O'Donoghue tubes, and others) at the end of the procedure is not a consensus. Many authors save the tubes only for revision cases, in narrow nasal cavities or in cases of canalicular stenosis^25^, ^26^.

Other factors favoring intubation, although without hard evidence, would be a prior episode of an acute dacryocystitis, bad flaps, excessive bleeding, inflammatory disease and small lacrimal sacs^4^.

**Figure 8:**
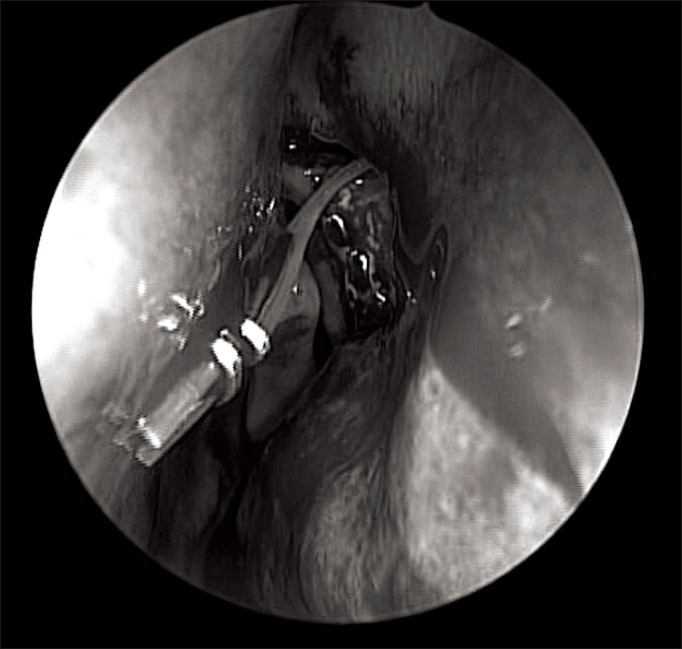
Notice the silicone tubes fxed with Ligaclip inside the nasal cavity.

When utilized, the tubes are inserted through the superior and inferior canaliculi and fixed inside the nose, being careful to leave a small gap in the external eye cantus. Inside the nose the surgeon can use size 100 connection clips, usually employed in neurosurgery and in vascular surgery to fix the two ends of the silicone tube (Figure 8). Another option for the endonasal fixation is to make knots. The excess of silicon is then removed. A piece of gelfoam may be placed around the tube in order to keep in place the previously made flaps. The silicone tube's dwelling time varies, but it should stay in for a least 4 weeks.

#### Postoperative care

Postoperative care is essentially the same used in any other endoscopic sinonasal surgery. Patients are instructed to sleep with high pillows and they must refrain from blowing their noses and doing vigorous physical activities for 10-14 days. Nasal flushings with saline solution are important and in some cases the patient must also add hydrocortisone drops for a few days. Antibiotic use is controversial in the literature^27^. Notwithstanding, it is frequently recommended to use oral antibiotics (amoxicillin/clavulanic acid) for 5-7 days. Ophthalmological drops with antibiotics and steroids are also prescribed for 14 days.

When a silicone tube is inserted, it is usually removed in the office after the first 4 weeks of post-op. The nasolacrimal system is then assessed by placing some fluorescein drops in the conjunctiva and monitor it through endoscopic view inside the nose. Granulation tissue, when present, must be removed in the office.

### Results and causes of surgical failure

In order to be a success, two goals must be achieved: anatomical patency and symptom alleviation. In other words, the patient must be asymptomatic and the lacrimal system must be patent, which is confirmed by nasal endoscopy (a positive fluorescein test). The mean success rate according to the criteria above in expert hands is higher than 90%-95% in primary cases of anatomical obstruction. In primary cases with functional obstruction, this rate drops down to 80%-85%. In revision cases, success rates, although high, are reduced^17^.

The main causes for surgical failure are listed on Table 2.

**Table 2:** Causes for failure of the endoscopic dacryocystorhinostomy.

1. Failure in locating the lacrimal sac
2. Insuffcient osteotomy
3. Granulation tissue, fbrosis or local synechia
4. Bone neogenesis
5. Insuffcient opening of the lacrimal sac

### Complications

The complication rate of endoscopic dacryocystorhinostomy in experienced hands are never higher than 2%. The rates hereby described are similar to the ones found in patients operated by the external approach.

The most important complications stem from the surgeon losing sight of the anatomical landmarks during the procedure, with consequent damage to neighboring structures^17^, ^28^. More frequently, the dissection is extended too posteriorly, damaging the drainage of the maxillary and frontal sinuses. Orbit penetration, with exposure of the orbital fat, bleeding and damage to the eye muscles may also happen. The key anatomical landmark used to avoid such complications is the unciform apophysis - it must be spotted early on during the procedure and represents the posterior limit for the dissection.

Other complications described included postoperative bleeding requiring nasal packing, CSF leaks and corneal and/or canalicular erosion. Special care must be observed in children during resection of the upper bone wall (lower skull base), in order to avoid CSF leaks.

## CONCLUSION

When properly indicated, dacryocystorhinostomy boasts a high success rate to treat epiphora, externally or through the endoscopic endonasal approach^10^, ^29^, ^30^. The advantages of the endoscopic approach are the magnificent view of the lacrimal sac in its entirety, no external scar, the preservation of the lacrimal pump through the eye's orbicular muscle, the possible lower intraoperative time and the lower morbidity. Moreover, other sinonasal disorders may be corrected in the same surgical procedure, such as nasal septum deviation and adenoid hypertrophy. Nevertheless, the endoscopic technology and associated instruments increase the cost of the procedure^29^. In many cases the concomitant external correction of problems associated with the superior and/or inferior canaliculi may become necessary. Thus, DCR is today a surgery which is carried out with excellent results by the ophthalmologist as well as by the rhinologist. The team work: ophthalmologist + otorhinolaryngologist, may bring about much benefit to the surgical treatment of patients with epiphora.
